# Microscopic colitis in Northern Ireland: an updated clinicopathological audit and assessment of compliance with European guidelines

**DOI:** 10.1111/codi.16254

**Published:** 2022-07-31

**Authors:** Michelle Moore, Helen G. Coleman, Patrick B. Allen, Maurice B. Loughrey

**Affiliations:** ^1^ Department of Cellular Pathology Royal Victoria Hospital, Belfast Health and Social Care Trust Belfast UK; ^2^ Centre for Public Health Patrick G. Johnston Centre for Cancer Research, Queen's University Belfast Belfast UK; ^3^ Department of Gastroenterology South‐Eastern Health and Social Care Trust Belfast UK

**Keywords:** colitis, endoscopy, microscopic colitis

## Abstract

**Aim:**

We previously reported the first population‐based study of the epidemiology of microscopic colitis in Northern Ireland. The aim of the current study is to provide updated data on incidence, diagnostic methods and clinicopathological associations, following dissemination of the previous report. A further aim was to compare the findings against relevant recommendations from the 2020 European guidelines.

**Method:**

Study cases were identified via the Belfast Health and Social Care Trust pathology laboratory system for new cases of collagenous colitis or lymphocytic colitis diagnosed from 2017 to 2020 inclusive. Demographic and clinical information was collated from electronic healthcare records.

**Results:**

Two hundred and seventeen new diagnoses of microscopic colitis were made between 2017 and 2020, comprising 89 (41%) collagenous colitis and 128 (59%) lymphocytic colitis. The overall incidence of microscopic colitis, expressed per 100,000 adult population, ranged from 7.6 to 11.5 (5.9 to 9.0 per 100,000 total population). The 2019 peak of 11.5 cases per 100,000 adult population represents a 71.6% increase in incidence compared with the mean incidence of 6.7 per 100,000 adult population from previous data for 2008–2016. There has also been a significant increase in number of cases diagnosed on separate sampling from the right and left colon (85% in 2019–2020 compared with 30% in 2008–2016; *p* < 0.001). Overall compliance with coeliac serology testing has improved, with 89% tested in 2017–2018 compared with 75% in 2008–2016.

**Conclusion:**

Clinicopathological communication has contributed to an increased incidence of microscopic colitis in Northern Ireland through better endoscopic diagnostic sampling and pathology coding practices. Coeliac serology testing has also improved, although continued clinical awareness is required of the need for coeliac serology testing in all patients diagnosed with microscopic colitis.

**What does this paper add to the literature?**

This paper is a follow‐up to the first epidemiological assessment of microscopic colitis in Northern Ireland, providing more complete data on incidence and assessing compliance with endoscopic sampling and coeliac serology testing, both of which are key aspects of the management of microscopic colitis outlined within recent European guidelines.

## INTRODUCTION

Interest in microscopic colitis (MC), comprising collagenous colitis (CC) and lymphocytic colitis (LC), has increased in recent years. Rising incidence of this disease accompanies improved clinical awareness, particularly in Europe and North America [1, 2, 3]. Whilst not life‐threatening, MC causes chronic, watery diarrhoea which can significantly impair quality of life. Symptomatic change in bowel habit may trigger referral along cancer diagnostic pathways and therefore this disease is encountered by both gastroenterologists and colorectal surgeons. Whilst remission can be achieved with medical therapy in the majority of patients following histological diagnosis [4, 5, 6, 7, 8, 9, 10], approximately 50% of cases follow a relapsing–remitting course [11].

We previously reported the incidence and clinicopathological associations of MC in a population‐based study in Northern Ireland, covering the period 2008–2016 [12]. This study highlighted a rising incidence of MC comparable with other studies but was hampered by incomplete pathology diagnostic coding procedures, resulting in likely underestimation of the true incidence of LC in particular. Clinically, well‐established associations with autoimmune diseases and certain drug groups were demonstrated. Of relevance to the United European Gastroenterology (UEG) and European Microscopic Colitis Group (EMCG) guidelines published in 2020 were two specific data items [13]. Firstly, whereas separate endoscopic sampling from the right and left colon is recommended when considering a diagnosis of MC, this was performed in only 30% of cases in our previous study, the remaining 70% of diagnoses being made on random or distal biopsies only. Secondly, given an increased incidence of coeliac disease in patients with either form of MC, guidelines recommend coeliac serology testing after any new diagnosis of MC. Our previous study indicated that coeliac serology testing was performed in 75% of study patients.

We therefore conducted a follow‐up audit of the Northern Ireland population, covering the years 2017 to 2020 inclusive. The years 2017 and 2018 account for the interventional period, during which we disseminated the findings from our previous study to clinicians, via presentations at regional gastroenterology and surgical meetings, and to pathologists, by direct e‐mail communication, to flag the publication and specifically to advise on appropriate SNOMED coding for such cases. The years 2019 and 2020 represent a postinterventional period. The primary aim was to provide updated data on the incidence of MC and diagnostic methods. A second aim was to compare selected data items, specifically endoscopic sampling method and performance of coeliac serology testing, against relevant recommendations from the 2020 European guidelines.

## METHOD

Study cases were identified through an archival SNOMED search performed within the laboratory information management system of the Belfast Health and Social Care Trust cellular pathology laboratory. This serves two of five trusts within Northern Ireland, providing secondary care to a total population of approximately 725,000 people, including an adult population (aged 18 years or older) of approximately 565,000 (the total population of Northern Ireland being 1.9 million). Incidence data were calculated per 100,000 of both the total population and adult population residing in the catchment area during the years 2017 and 2020 (https://www.nisra.gov.uk/).

Cases of MC, CC or LC diagnosed from 2017 to 2020 inclusive were searched for using the closest available SNOMED codes ‘microscopic colitis’ (SNOMED code D541725), ‘collagenous colitis’ (D544130) and ‘lymphocytic–plasmacytic colitis’ (D541710). Note that no specific code is available for ‘lymphocytic colitis’ but, following our previous study, recommended SNOMED codes for MC were disseminated to all pathologists in mid‐2018. No pathology slide review was conducted but all diagnoses were made by one of a team of specialist gastrointestinal pathologists, usually on routine haematoxylin and eosin (H&E)‐stained slides without the use of ancillary stains, such as CD3 immunohistochemistry or collagen stains such as Masson Trichrome or Van Gieson. These stains were typically reserved for borderline cases, reflecting current guidance and routine practice [13]. Cases with a previous established diagnosis of MC were excluded. As in our previous study, the diagnostic term ‘incomplete microscopic colitis’ was not in clinical use during the study period and it is likely that such cases will not have had any MC SNOMED code added and not have been captured.

In order to allow direct comparison with the 2008–2016 cohort, similar patient characteristics were collected as before. Ethical approval was not required for this clinicopathological audit. Information collected included age at diagnosis, sex and endoscopic information regarding the procedure performed (colonoscopy or sigmoidoscopy) and the nature of diagnostic biopsy sampling. For this purpose, the right colon was considered proximal to the splenic flexure and the left colon represented the splenic flexure and descending or sigmoid colon. Incidence data for 2019 and 2020 were analysed separately, given the likely impact on elective endoscopy services of the SARS‐CoV‐2 (COVID‐19) global pandemic [14]. Follow‐up information was restricted to whether or not coeliac serology was performed prior to/following diagnosis of MC. Chi‐square tests were applied to compare proportions between timeframes using Stata version 14.2 (StataCorp).

## RESULTS

**FIGURE 1:**
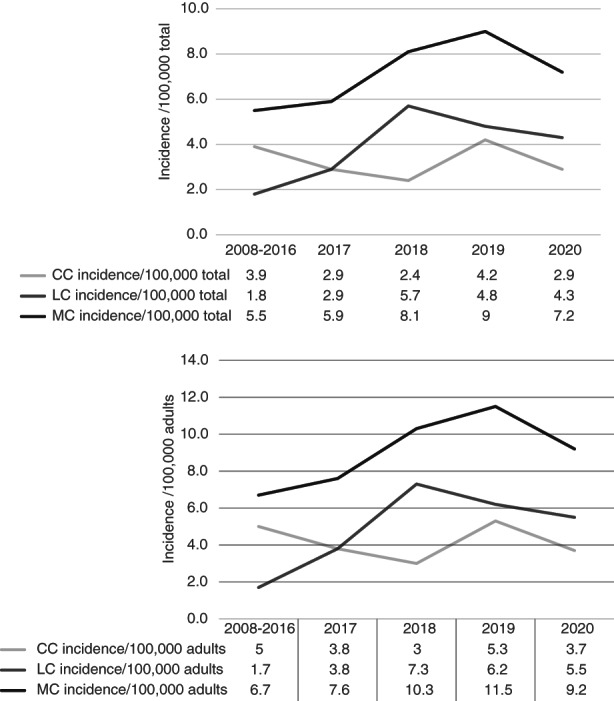
Mean annual incidence of microscopic colitis (MC), collagenous colitis (CC) and lymphocytic colitis (LC) from 2008–2016 [12] compared with annual incidences thereafter, expressed per 100,000 total population and per 100,000 adults

A total of 217 new diagnoses of MC were made between 2017 and 2020, comprising 89 diagnoses of CC and 128 of LC. Overall MC incidence, expressed per 100,000 total population, ranged from 5.9 to 9.0, with a peak incidence in 2019 of 9.0, comprising an incidence for CC of 4.2 and for LC of 4.8 during this year (Figure 1). This corresponds to an incidence range between 2017 and 2020 of 7.6 to 11.5 per 100,000 adult population, with a peak incidence in 2019 of 11.5, comprising an incidence for CC of 5.3 and an incidence for LC of 6.2. This 2019 peak represents a 71.6% increase in incidence compared with the mean incidence of 6.7 per 100,000 adults from our previous 2008–2016 data [12], almost exclusively due to a marked increase in reported LC cases.

**FIGURE 2:**
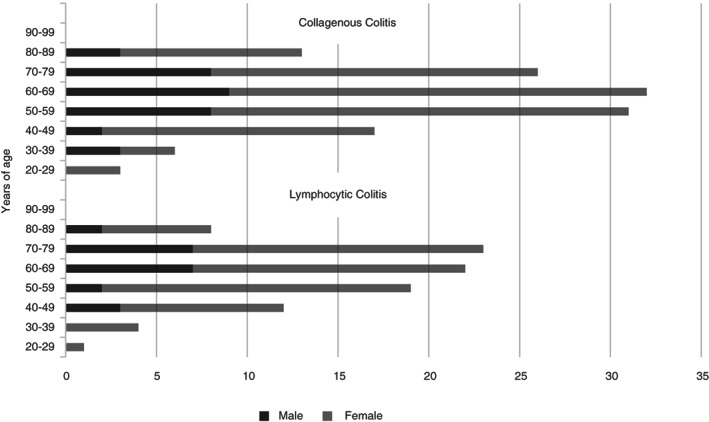
Relationship between age at time of diagnosis and sex in all cases of lymphocytic and collagenous colitis diagnosed from 2017–2020 (*n* = 217)

Demographic information collected on all cases is summarized in Figure 2. Overall, these data are very similar to those for the 2008–2016 cohort [12]. A strong female predominance is evident for both CC and LC (76% and 74% of cases, respectively) with a median age of diagnosis of 64 years for CC and 62 years for LC, albeit with a wide age range for each diagnosis. No cases of paediatric (<18 years of age) MC were identified.

**FIGURE 3:**
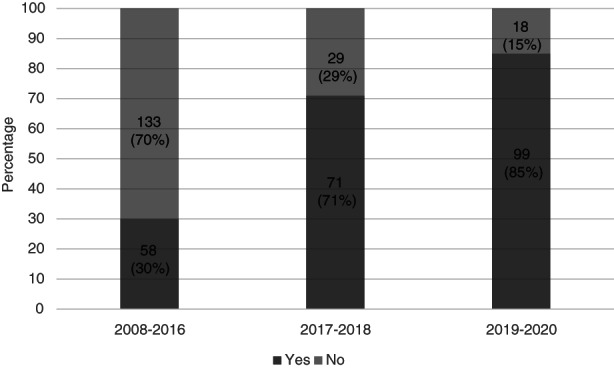
Percentages of cases of microscopic colitis in three time periods which were diagnosed using two or more endoscopic specimens

**FIGURE 4:**
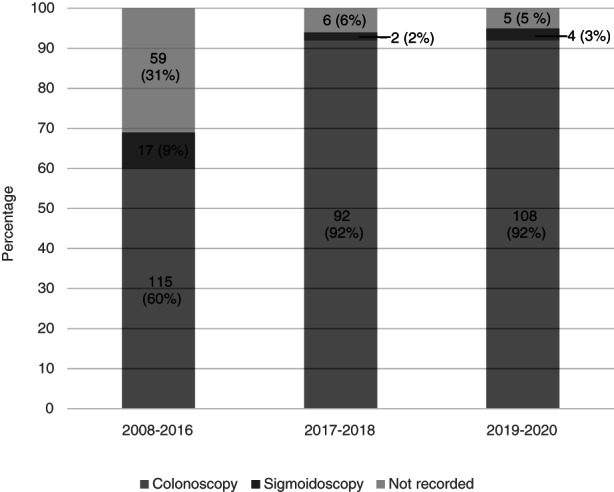
Percentages of cases of microscopic colitis in three time periods diagnosed using colonoscopy and sigmoidoscopy

In the interventional period (2017–2018), 71 of 100 cases (71%) were diagnosed from two or more separately labelled biopsy specimens, usually representing the right and left colon (Figure 3). This upward trend continued in 2019–2020, when 99 of 117 cases (85%) were diagnosed from at least two separately labelled specimens. This was significantly higher than during 2008–2016, in which only 30% of cases had separate right and left colonic sampling (*p* < 0.001) [12]. During 2017–2018 and 2019–2020 at least 92% of cases of MC were diagnosed by colonoscopy, with 2%–3% by sigmoidoscopy (procedure was unrecorded in 5%–6%) (Figure 4). In comparison, during the period 2008–2016 at least 60% of diagnoses were made after colonoscopy and at least 9% after sigmoidoscopy (procedure was unrecorded in 31%).

**FIGURE 5:**
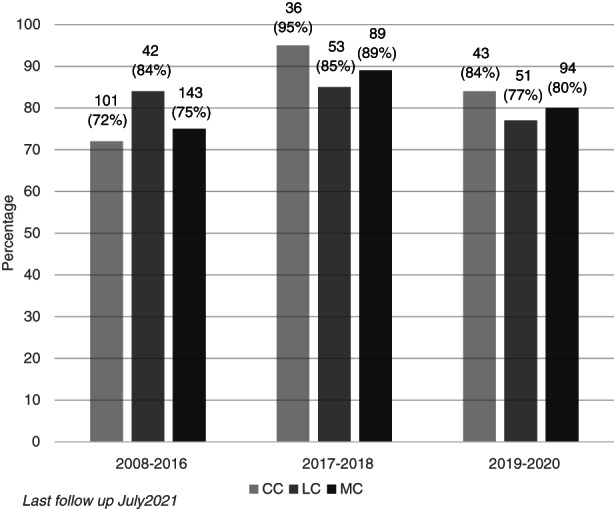
Percentages of patients with microscopic colitis (MC), lymphocytic colitis (LC) and collagenous colitis (CC) diagnosed in three time periods who had coeliac serology performed

Of the 2017–2018 cohort, 89% of patients with MC (95% with CC and 85% with LC) had coeliac serology performed at any time in their clinical history (before or after diagnosis) (Figure 5). Testing was lower in the 2019–2020 cohort. At the time of last follow‐up (July 2021), 80% of patients diagnosed with MC (84% with CC and 77% with LC) had coeliac serology performed at any time in their clinical history.

## DISCUSSION

This updated population‐based audit of MC diagnoses in Northern Ireland reinforces previous, well‐recognized clinicopathological features of female predominance, median age of diagnosis in the seventh decade and a wide age range [12]. Following feedback on good practice points from our previous study to clinicians and pathologists, the annual incidence of MC peaked at 9.0 per 100,000 total population (11.5 per 100,000 adult population) in 2019. As this increase is largely due to the number of LC cases, it is likely due primarily to better pathology coding of LC rather than a true increase in incidence. The increase probably also reflects generally greater awareness of this diagnosis amongst clinicians and pathologists, and specifically the need to sample endoscopically normal colonic mucosa in patients presenting with chronic, nonbloody diarrhoea. Such practice is important to both gastroenterologists and colorectal surgeons, as patients with MC may follow medical or surgical pathways. The incidence in this population remains lower than the overall pooled incidence rate of MC of 11.4 per 100,000 person years, calculated recently from 22 studies which provided population‐based data, many from northern Europe [13]. This suggests that there are further cases to be identified in the Northern Ireland population, requiring further education amongst healthcare groups, including primary care and colorectal surgeons. The much lower incidence of MC in 2020 of 7.2 per 100,000 total population, compared with 9.0 in 2019, most likely reflects emergence of the SARS‐CoV‐2 (COVID‐19) global pandemic and its impact on elective endoscopy services [14].

Despite an absence of high‐quality evidence, separate sampling and submission of right and left colonic biopsies is a strong recommendation in current British and European guidelines [13, 15]. Although the histological changes of both LC and CC are typically uniform throughout the colon, normal lamina propria cellularity is higher in the right colon and there is more often mild nonspecific subepithelial collagen deposition in the sigmoid colon and rectum [16, 17]. For these reasons, awareness of the biopsy site can help pathological interpretation, especially in borderline cases. The current study further demonstrates the potential value of clinical education and clinicopathological communication, with submission of separate right‐ and left‐sided endoscopic biopsies increasing from 30% (2008–2016 cohort) to 85% (2019–2020 cohort). It is possible that a change in practice from sigmoidoscopy to greater use of colonoscopy contributed to this finding of reduced single‐site biopsy sampling, with growing acceptance of the need for patients with chronic diarrhoea to be investigated by full colonoscopy if possible [15]. However, although our data regarding nature of endoscopy procedures between study time periods are incomplete, available data suggest that the marked increase in separate right and left colonic sampling reflects a true change in biopsy regime. This enhanced diagnostic sampling may also have contributed to the increased incidence observed, with pathologists able to offer more confident diagnoses on more precisely labelled specimens.

Coeliac disease serology testing following a diagnosis of MC is a strong recommendation in recent European guidelines, given good evidence of an association with both LC and CC [13]. In one large prospective study, the incidence of coeliac disease was 3.3% in patients with MC compared with 0.4% in controls [18]. Large cohort studies and one pathology registry study document incidence rates from 2%–4% [19, 20, 21]. The current study represents improved overall testing of coeliac serology in MC, rising from 75% in 2008–2016 to 89% in the 2017–2018 cohort. This improvement was due almost exclusively to a 23% increase in testing patients with CC, suggesting less prior awareness amongst clinicians of the association between coeliac disease and CC. It should be noted that this improvement pre‐dates publication of the UEG/EMCG guidelines in 2020 and most likely relates to educational feedback from our previous study. Less follow‐up is available for the 2019–2020 cohort, due to the limited time elapsed from diagnosis to last follow‐up, precluding confident comparison with older cohorts, but there appears to be a reduction in coeliac serology testing in these more recent years (80% at last follow‐up), indicating a need for continued clinical education in this regard. The SARS‐CoV‐2 pandemic may also have affected testing, with reduced access in 2020 to follow‐up appointments and routine blood tests. Perhaps lacking from current guidelines, and warranting further consideration, is specification of a suitable timeframe within which coeliac serology testing should be performed to provide a reliable assessment of current coeliac status in those diagnosed with MC.

To conclude, clinicopathological communication is likely to have contributed to an increased incidence of MC in Northern Ireland, most demonstrably through better endoscopic diagnostic sampling, alongside improved pathology coding practices. Coeliac serology testing has also improved, although continued clinical awareness is required of the need for coeliac serology testing in all patients diagnosed with MC.

## AUTHOR CONTRIBUTIONS

All authors have made substantial contributions to all of the following: (1) the conception and design of the study, or acquisition of data, or analysis and interpretation of data, (2) drafting the article or revising it critically for important intellectual content, (3) final approval of the version to be submitted. Specific contributions are outlined below: MM, acquisition of data and analysis, drafting the article, final approval of the submitted version; HGC, interpretation of data, statistical analysis, article revision, final approval of the submitted version; PBA, interpretation of data, article revision, final approval of the submitted version; MBL, conception and design of the study, interpretation of data, final approval of the submitted version, guarantor.

## FUNDING INFORMATION

No funding received.

## CONFLICT OF INTEREST

None.

## Data Availability

Supporting data are not publicly available in order to protect patient privacy. Anonymized data are available upon reasonable request. Population data for Northern Ireland are available at https://www.nisra.gov.uk/.
